# Some Functional Properties of *khambir*, an Ethnic Fermented Cereal-Based Food of Western Himalayas

**DOI:** 10.3389/fmicb.2019.00730

**Published:** 2019-04-24

**Authors:** Papan K. Hor, Mousumi Ray, Shilpee Pal, Kuntal Ghosh, Jyoti P. Soren, Smarajit Maiti, Debabrata Bera, Somnath Singh, Sanjay Dwivedi, Miklós Takó, Pradeep K. DasMohapatra, Keshab C. Mondal

**Affiliations:** ^1^Bioinformatics Infrastructure Facility Center, Department of Microbiology, Vidyasagar University, Midnapore, India; ^2^Department of Biological Sciences, Midnapore City College, Paschim Medinipur, India; ^3^Department of Biochemistry and Biotechnology, Cell and Molecular Therapeutics Laboratory, Oriental Institute of Science and Technology, Midnapore, India; ^4^Department of Food Technology, Jadavpur University, Kolkata, India; ^5^Division of Nutrition, Defense Institute of Physiology and Allied Sciences, New Delhi, India; ^6^Defence Research Laboratory (Defence Research and Development Organisation), Tezpur, India; ^7^Department of Microbiology, Faculty of Science and Informatics, University of Szeged, Szeged, Hungary

**Keywords:** fermented *khambir*, antimicrobial, docking, antioxidant, antitoxicant activity

## Abstract

Traditional leavened wheat-based flat bread *khambir* is a staple food for the high-altitude people of the Western Himalayan region. The health promoting abilities of two types of *khambir*, yeast added *khambir* (YAK) and buttermilk added *khambir* (BAK), were evaluated. A group of microbes like yeast, mold, lactic acid bacteria (LAB), and *Bifidobacterium* sp. were abundant in both *khambir* but in varied proportions. Both are enriched with phenolics and flavonoids. The aqueous extracts of both breads strongly inhibited the growth of enteropathogens. Molecular docking experiments showed that phenolic acid, particularly *p*-coumaric acid, blocked the active sites of β-glucosidase and acetylcholine esterase (AChE), thereby inhibiting their activities. YAK and BAK showed antiradical and antioxidant activity ranging from 46 to 67% evaluated using 2,2-diphenyl-1-picrylhydrazyl (DPPH), 2,2-azino-bis-3-ethylbenzothiazoline-6-sulfonic acid (ABTS), and ferric reducing/antioxidant power (FRAP) assays. The aqueous extract of both *khambir* samples protected the arsenic toxicity when examined under an *in situ* rat intestinal loop model study. The arsenic induced elevated levels of superoxide dismutase (SOD), catalase (CAT), reduced glutathione, lipid peroxidation (LPO) and DNA fragmentation, and transmembrane mitochondrial potential was alleviated by *khambir* extract. These results scientifically supported its age-old health benefit claims by the consumer at high altitude and there are enough potentialities to explore *khambir* as a medicinal food for human welfare.

## Graphical Abstract

Health supportive properties of *khambir*.

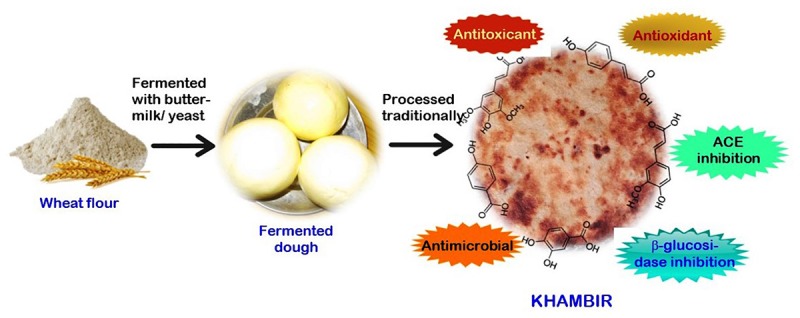

## Introduction

Traditional fermented foods have greater preference in certain communities due to typical characteristics such as flavor, color, and texture (Mondal et al., 2016). Most of the fermented foods contain an increased amount of health beneficial nutraceuticals, bioactive components, and good microbes compared to their unfermented substrate (Tamang et al., 2016). Due to age-old safety and beneficial experiences, scientists have been focused on exploring their nutrient profile, wild microbial resources, and therapeutic components, standardizing process parameters for the welfare of mankind.

**FIGURE 1:**
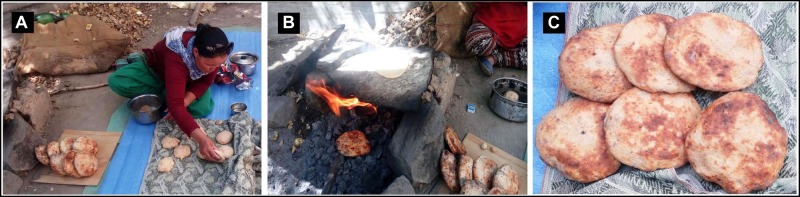
Traditional process of *khambir* preparation. After overnight incubation of wheat flour and starter (yeast or buttermilk), the fermented dough is divided, and ball shaped by hand **(A)**. The handmade round-shaped dough is baked over a hot stone and then under direct fire **(B)**. The final cleaned and polished brown bread is ready for consumption **(C)**.

Wheat-based handmade flat bread is a traditional and popular staple food, particularly in the Middle East, North Africa, and Central Asia (Parimala and Sudha, 2015). Several types of wheat-based flat bread in Middle Eastern households were documented by Al-Dmoor (2012) and in India by Mir et al. (2014) and Parimala and Sudha (2015). India is the second largest producer of wheat and native people prepare a variety of flat breads with different tastes and textures. Different ingredients like rice/a rice–wheat mix (e.g., *selroti*), finger millet (e.g., a*mbali*), or wheat (e.g., *nan*, *bhaturu* or *bhatooru*, *chilra, seera*, etc.) are commonly used for the preparation of delicious fermented flat bread in India. There are exceptionally few places where yeast, curd, or butter milk is added to wheat flour, fermented overnight, and then baked using traditional methods. *Tagi Khambir* or commonly called *khambir* is a “browned sourdough bread” – a very popular staple food at the high altitudes of the Himalayas like in Leh – Ladakh region of Jammu and Kashmir state, India (Angchok et al., 2009) and in a few places in Tibet and China (Tamang, 2010). According to the belief of the native people, this leavened bread can protect them against harsh environmental stresses (extreme cold at around -25°C during winter, strong wind, and low humidity) and provide adequate energy and mouth feeling (Angchok et al., 2009). The culinary practice is also unique and seems to be an inherited food culture of the Indus valley civilization. Rural women have the required knowledge of the proper art of baking this bread. Cleaned wheat flour is mixed with an adequate amount of salt, water, and buttermilk or yeast powder (marketed Baker’s yeast). Then the dough is wrapped with a clean cloth and kept in a traditional kitchen overnight. Layers of cloth are wrapped over the container to maintain the temperature. The next day, the fermented dough is divided and hand-shaped into small ball-like structures (each having the weight around 200 g). It is then baked initially on hot stones and finally, directly in a fire made of wood or cow dung. It is finished off on the embers inside the fire, using edible oil (Figure 1). It can be stored at room temperature for more than a week. These traditional flat breads are gaining popularity among outsiders due to the rapid growth of “village tourism,” “home stay,” or “ethnic food tourism” in these regions (Tamang, 2010).

Although the native people believe in the health benefits of *khambir*, surprisingly no such study has been conducted to validate this. Considering this, we examined the health benefits (antimicrobial, trypsin, acetylcholine, and β-glucosidase inhibitory activities, antioxidant, and detoxicant activity) of homemade *Tagi Khambir*. Moreover, the ameliorative role of its extract was tested against arsenic (a globally recognized environmental pollutant and Gr. A carcinogen) induced toxicity, in an *in situ* loop model study of rat intestine, to prove its detoxification activity.

## Materials and Methods

### Chemicals

All the chemicals used in this study are of analytical grade and was procured from standard companies.

### Sample Collection and Preparation

*Khambir* samples were collected from households in two villages (*Sabu* and *Pheyang* which are about 10 km away from the town of Leh) in the Leh district, Jammu and Kashmir state, India. Three types of preparation, *viz*., standard white wheat bread (unfermented, used as control), yeast (marketed Baking yeast) added *khambir* (YAK), and buttermilk added *khambir* (BAK) were collected. Then the samples were transferred into a sterile container and transported to the laboratory through an ice box. Bread samples were dried in a food dryer at 55°C for 10 h, and then dissolved into sterile distilled water (0.1%, w/v) by homogenization and centrifuged at 2000 *g* for 10 min. The supernatant was used as a food extract for further studies.

### Microbiological Analysis

The quantity of the prevalent group of microbes in the food samples (direct sample) was enumerated on the basis of colony-forming units (cfu). The counts of different bacterial group were performed based on their colony morphology and color in various selective and differential agar media. Briefly, 10 g of the raw sample was mixed with a 100 ml of phosphate buffer saline (pH 7.2) and used as stock for the microbial count. The group of lactic acid bacteria (LAB) and *Bifidobacterium* sp. were cultivated in Rogosa SL agar (supplemented with 0.132% acetic acid) and Bifidobacterium agar supplemented with Bifidobacterium Selective Supplement (HiMedia, FD285), respectively, and plates were incubated in a CO_2_ incubator (5% CO_2_), at 37°C (Adak et al., 2013). All of the luxuriant growing colonies were enumerated for the above-mentioned bacteria. Total aerobic bacteria were quantified using Plate Count Agar (PCA) media and incubated at 37°C (Adak et al., 2013). Yeast and mold were enumerated by using yeast and mold agar, and Potato Dextrose Agar (PDA) media, respectively, and incubated at 30°C. The mycelial and round convex colonies were recorded for the mold and yeast counts, respectively. MacConkey agar and Salmonella differential agar were used for the determination of *Escherichia coli* and *Salmonella* sp., respectively. The plates were incubated at 35°C for 24 to 48 h. The pink red with bile precipitated colonies grown on the MacConkey agar were enumerated for *E. coli*. Moreover, the colorless and pink red colonies were counted for *Salmonella* sp. For *Vibrio* sp. enumeration, the yellow and bluish green color colonies grown on Thiosulfate Citrate Bile salt Sucrose (TCBS) agar base were selected.

### Estimation of Total Phenolic Content

Total phenolic content was determined by the Folin–Ciocalteu method as described elsewhere. One milliliter of bread extract (100 mg/ml), 5 ml of diluted Folin–Ciocalteu phenol reagent (1:10 distilled water), and 4 ml of sodium carbonate solution (7%, w/v) were added sequentially. Soon after mixing the reactants, the test tubes were placed in the dark for 1 h and the absorbance was recorded at 725 nm against a reagent blank. The total content of phenolic compounds in extracts was expressed as a gallic acid equivalent (GAE) and mg/g of the dry sample.

### Extraction of Phenolics and Chromatographic Analysis

The samples (300 mg) were extracted with 3 ml of methanol/water (80/20, v/v), for 10 min by sonication at room temperature. After centrifugation at 8000 rpm for 5 min, the supernatant was removed, and the extraction was repeated two times in a similar way. The combined supernatants were evaporated to dryness by centrifugal evaporation. The residues were dissolved in 400 μl of methanol/water (80/20, v/v) and filtered through a 0.2 μm PTFE membrane filter. A 20 μl of the final solution was injected into the HPLC system.

Phenolic compounds were separated on a LUNA-PFP (2) (3 μm, 150 mm × 4.6 mm) column thermostated at 35°C. Mobile phase A consisted of methanol/water (10/90, v/v) containing 0.1% acetic acid, while methanol containing 0.1% acetic acid served as mobile phase B. The gradient elution was performed as follows: 0.0 min, 5% B; 6.5 min, 25% B; 30.5 min, 37% B; 35.0 min, 55% B; 37.0 min, 95% B; 44.0 min, 95% B; 45.0 min, 5% B and 50.0 min; 5% B for re-equilibration of the column. The flow rate was adjusted to 0.7 ml/min. The injection volume was 20 μl. Phenolic compounds were monitored at 280 and 320 nm. For quantification, standards (Sigma–Aldrich, United States) of two subgroups of phenolic acid, viz., hydroxybenzoic acid (protocatechuic acid and *p*-hydroxy-benzoic acid) and hydroxycinnamic acid (*p*-coumaric acid, ferulic acid, and sinapic acid) were employed.

### Estimation of Total Flavonoids Content

For the estimation of the flavonoid, the bread extract of 0.5 ml was mixed with 0.1 ml of 5% C_4_H_4_O_6_KNa⋅4H_2_O [potassium sodium L-(+) tartrate]. After 5 min, 0.1 ml of 10% aluminum chloride was added to the mixture and made up to 3 ml using distilled water. After incubation at room temperature for 1 h, the absorbance of the reaction mixture was measured at 430 nm against a blank that contained 0.1 ml of distilled water in place of aluminum chloride. The total flavonoid content was expressed (mg/g) as a quercetin equivalent.

### Bioactivities of *Khambir* Sample

#### Antimicrobial Activity

Antimicrobial activity of *khambir* extract was tested (disc diffusion method, 6 mm) against different strains of human pathogens (some were locally isolated and characterized, and some were type cultures) like *Aeromonas hydrophila* SBK1, *Salmonella typhi* B3274, *S. typhi* E1590, *S. typhi* MTCC 734, *Shigella dysenteriae* 4717, *Shigella sonnei* RS 1, *Staphylococcus aureus* MB 13, *Streptococcus faecalis* MB 15, *Micrococcus luteus* ATCC 9341, and *Vibrio harveyi* MTCC 7954. The aqueous extract of the *khambir* samples (0.1%, w/v) was filter sterilized and 50 μl of the sample was tested against the above-mentioned pathogenic bacteria, which were spread onto the Mueller–Hinton agar (HiMedia, India) media and grown at 37°C for 24 h. Tetracycline (30 μg) was used as positive control. Thereafter inhibition zones that formed around the disc were measured and compared with an antibiotic.

#### Effect of *Khambir* Extract on the Activity of Some Health Indicator Enzymes

The bread sample was mixed with 10% diaion HP 20 resin (Sigma) under shaking for 30 min on a magnetic stirrer. Then the flask contents were eluted with 20 ml methanol. The collected methanol fractions were evaporated in a rotary evaporator (EYELA, Japan) and the residue was dissolved in DMSO and stored at -20°C for further analysis.

#### β-Glucosidase Inhibition Assay

The assay was performed according to the plate assay method as described by Pandey et al. (2013). Briefly, a 10 ml agar solution was prepared by mixing 0.07 g of agar powder in 0.1 M acetate buffer (pH 5.0) and dissolved at 80–100°C; followed by the addition of 1.2 ml of FeCl_3_ (0.5%, w/v) solution and 40 μl (0.01 U/ml) of β-glucosidase (Sigma–Aldrich, source-almonds). This mixture was poured onto petri dishes and allowed to settle and firm up. The bread extract of 5 μl was spotted on the surface of the agar plate. Similarly, conduritol β-epoxide (Sigma), an irreversible inhibitor (0.75 μg), was used as a positive control and DMSO without extract was used as a negative control. The plates were incubated at room temperature for 15 min for an interaction between the enzyme and inhibitor. Later on, 7.0 ml (0.2%, w/v) of esculin (Himedia, India), the specific substrate for β-glucosidase, was floated on the surface of an agar plate and again incubated at room temperature for 30 min. Clear zones (CZs) were measured and compared to express the percentage (%) of enzyme activity or inhibition.

#### Determination of Trypsin Inhibition

The trypsin activity was assayed by the casein digestion method (Tripathi et al., 2011). Briefly, 1 ml of enzyme (SRL, India; Bovine pancreas, 1000 U/mg, 0.1 mg/ml) was incubated alone or with the bread extract for 20 min followed by the addition of 3.0 ml of 1% casein (in 100 mM Tris-HCl buffer; pH 8.0) at 37°C for 20 min. The reaction was stopped by the addition of 3.0 ml of 10% (w/v) trichloroacetic acid (TCA). The mixture was then centrifuged at 10,000 *g* and absorbance of the supernatant was measured at 280 nm to estimate the released tyrosine. One unit of trypsin activity was defined as the amount of enzyme that liberates 1.0 μg of tyrosine min^−1^ml^−1^ under standard assay conditions.

#### Acetylcholine Esterase (AChE) Inhibition Assay

The acetylcholine esterase (AChE) inhibitory activity of the bread extract was evaluated following the method of Elumalai et al. (2015). Briefly, 0.1 M phosphate buffer (pH 8.0, 150 μl), food extract solution (10 μl), and enzyme solution (earthworm head extract, 20 μl) were mixed and incubated for 15 min at 25°C; 10 μl of DTNB (10 mM) was then added. The reaction was then initiated by the addition of substrate (10 μl of acetyl thocholine, 14 mM solution). The formation of the colored product was measured at 410 nm after 10 min of incubation. One unit of AChE activity was defined as the amount of enzyme that liberates 1.0 μg of choline min^−1^ml^−1^ under standard assay conditions.

#### *In silico* Molecular Docking Experiment

For the molecular docking study, an X-ray crystallography structure of AChE (PDB ID: 1FSS) with a resolution of 3.0 Å and β glucosidase (BG) (PDB ID: IOGS) with a resolution of 2.0 Å were retrieved from the Protein Data Bank (PDB). Active sites or cavities of the selected target proteins were identified using the CAStp server^1^. The target proteins were developed for docking by deleting water and adding polar hydrogen. The structure of the positively correlated phenolic compound (*p*-coumaric acid) was downloaded from the NCBI PubChem database^2^ and converted into pdb (.pdb) format using Open Babel (O’Boyle et al., 2011). Then docking was performed by using Autodock Tool (version 1.5.6) while PyMol (version 2.0) was used for visualization of the docked structure (Sanner, 1999; Morris et al., 2009).

### Assay of *in vitro* Antioxidant Activity

#### DPPH (2,2-Diphenyl-1-Picrylhydrazyl) Free Radicals Scavenging Test

The water extraction of *khambir* (150 μl) with a concentration of 100 mg/ml was mixed with 37.5 μl methanolic 2,2-diphenyl-1-picrylhydrazyl (DPPH) (0.75 mM) solution. DPPH without *khambir* extract served as a control. After 20 min of incubation, absorbance was measured at 517 nm (Ghosh et al., 2015).

$$\mathrm{DPPH scavenging activity}(\%)=(A_{\mathrm{control}}-A_{\mathrm{sample}}/A_{\mathrm{control}})\times 100$$

where *A_Sample_* is the absorbance of the sample and *A_control_* is the absorbance of the control.

#### ABTS (2,2-Azino-Bis-3-Ethylbenzothiazoline-6-Sulfonic Acid) Assay

2,2-Azino-bis-3-ethylbenzothiazoline-6-sulfonic acid radical-scavenging activity was assayed, following the method of Halder et al. (2014), with necessary modifications. The mixture (1:1 ratio) of ABTS (7.0 mM) and potassium persulfate (2.45 mM) was incubated at 25°C overnight before use. The working solution was prepared by diluting the stock solution with methanol to reach an absorbance of 0.7 ± 0.02 at 734 nm (A_control_). For measurements, 0.9 ml of the ABTS/ persulfate mixture and 0.1 ml of aqueous extract of *khambir* were mixed and absorbance was taken immediately after 15 min at 734 nm. The radical-scavenging activities (%) in both cases were calculated as follows:

$$\mathrm{Antioxidant activity}\;(\%)=(A_{\mathrm{control}}-A_{\mathrm{sample}}/A_{\mathrm{control}})\times 100$$

where *A_Sample_* is the absorbance of the sample and *A_control_* is the absorbance of the control.

#### Measurement of Ferric Reducing/Antioxidant Power (FRAP)

The reducing power of the *khambir* extract was measured (Yen and Chen, 1995) by mixing it with an equal volume of phosphate buffer (0.2 M, pH 6.6) and then incubating it at 50°C for 20 min with potassium ferricyanide (1%, w/v). The reaction was stopped by addition of TCA (10%, w/v) followed by centrifugation at 3000 rpm for 10 min. The supernatant was mixed with distilled water and ferric chloride (0.1%, w/v) solution, and the absorbance was measured at 700 nm. The reducing power (%) was calculated using the same equation used for DPPH or ABTS.

#### *In situ* Intestinal Loop Model Study to Assess Antitoxic Effect of *Khambir*

Inbreed male albino rats (150 ± 10 g) were used and fed rat specific standard food for 2 weeks prior to the experiment with Vidyasagar University Animal Ethical clearance (ICE/7-8/6-8/16 dt. 26.08.2016).

An *in situ* intestinal loop experiment was conducted as per the method described by Acharyya et al. (2015). Under anesthetize condition (by intramuscular injection of Ketamine-HCl, 22–24 mg/kg body wt.), the small intestinal portion was exposed sparingly from the abdominal cavity through a small cut on the cutaneous and abdominal muscle layers. In the small intestine, four loops (each having 2.0–2.5 inch in length) were created by creating five knots with a sterile cotton thread. These loops were filled (1 ml) sequentially by a syringe with a saline (control), aqueous extract of *khambir* (100 mg/ml), sodium arsenite (NaAsO_2_, 250 mM), and an aqueous mixture of *khambir* and sodium arsenite, respectively. The intestine was carefully placed back in its original location and the cut site was stitched up. After 24 h, animals were again anesthetized and euthanized by cervical dislocation. The intestinal portion was removed quickly, cleaned, and immediately perfused with ice-cold saline (0.85% sodium chloride). Epithelial cells in the inner layer were scraped out from different demarcated locations of the intestine using a Teflon scrapper and homogenized in ice-cold buffer (phosphate buffer, 0.1 M, pH 7.4). The homogenate was initially centrifuged at 3000 rpm for 10 min at 4°C in a Remi Cooling Centrifuge (C-24 DL) to separate the nuclear debris. The aliquot obtained was again centrifuged at 10,000 rpm for 20 min at 4°C to obtain the post-mitochondrial supernatant, which was used as a source of various enzymes. The protein content of the homogenate was estimated by the Lowry et al. (1951) method, using bovine serum albumin as standard.

#### Estimation of Superoxide Dismutase (SOD) Activity

The activity of superoxide dismutase (SOD) was measured following the method of Marklund and Marklund (1974). A reaction mixture was prepared comprising of 2.875 ml Tris–HCl buffer (50 mM, pH 8.5), 100 μl tissue homogenate, and pyrogallol (24 mM in 10 mM HCl), and the total volume was made 3.0 ml. The activity of the enzyme was measured at 420 nm and the unit (U) was expressed in units/mg protein. One unit of enzyme activity was defined as inhibition of the 50% auto-oxidation of pyrogallol, and calculated as:

$$\begin{array}{c} \mathrm{SOD}\;(\mathrm{units}/\mathrm{mg protein})=(\Delta \mathrm{OD sample}\times \mathrm{OD blank}\times 100)\\ \;/(\Delta \mathrm{OD sample}\\ \;\times 50\times \mathrm{Vol. of sample}). \end{array}$$

#### Estimation of Catalase (CAT) Activity

Catalase (CAT) activity was measured following the method of Aebi (1984). For assay, a reaction mixture was prepared with 2.0 ml phosphate buffer (0.1 M, pH 7.4), 0.05 ml of tissue homogenate, and 0.95 ml hydrogen peroxide (0.019 M) and the total volume was 3.0 ml. The activity of the enzyme was measured by taking absorbance at 240 nm. The CAT activity (U) was calculated in terms of nmol H_2_O_2_ consumed/minute/mg protein, with the help of the following formula:

$$\begin{array}{c} \mathrm{Catalase}\;(\mathrm{units}/\mathrm{mg of protein})=(\Delta \mathrm{OD}/\min\times \mathrm{Vol. of assay})\\ \;/(0.081\;\mathrm{of vol. of ensyme}\\ \;\mathrm{solution}\times \mathrm{protein content}). \end{array}$$

#### Estimation of Reduced Glutathione (GSH)

The method of Jollow et al. (1974) was adopted to measure the GSH level in the tissue. Briefly, 1.0 ml of sulfosalicylic acid (4%) was mixed with 1.0 ml of tissue homogenate. The sample was incubated for at least 1 h at 4°C and then centrifuged at 1500 rpm for 15 min at 4°C and used as the tissue mixture. The assay mixture contained 2.2 ml phosphate buffer (0.1 M, pH 7.4), 0.4 ml tissue mixture, and 0.4 ml of 5, 5’-dithiobis-2-nitrobenzoic acid, (DTNB, 10 mM) and absorbency was measured at 412 nm. The GSH content was calculated as μmol DTNB conjugate formed/g tissue using a molar extinction coefficient of 13.6 × 10^3^M^−1^ cm^−1^ with the help of the following formula:

$$\begin{array}{c} \mathrm{GSH}=(\Delta \mathrm{OD}/\min\times \;\mathrm{Vol. of assay}\times 100)\\ \;/(1.36\;\mathrm{of mole GSH conjugate}/\mathrm{g tissue}) \end{array}$$

#### Measurement of Lipid Peroxidation (LPO)

The level of membrane lipid peroxidation (LPO) was assayed following the method of Wright et al. (1981) with some modifications. The reaction mixture comprised of 1.0 ml cell homogenate, 1.0 ml of TCA (10%), and 1.0 ml TBA (0.67%). Then all the tubes were kept in a boiling water bath for 45 min. The tubes were then cooled at room temperature and centrifuged at 5000 rpm for 10 min. The optical density of the supernatant was measured at 532 nm. The level of LPO was measured with respect to malondialdehyde (MDA) formation and results were expressed as the mmol MDA formed/g tissue using a molar extinction coefficient of 1.56 × 10^5^M^−1^ cm^−1^, with the help of the following formula:

$$\mathrm{LPO}=(\mathrm{Vol. of assay}\times \mathrm{OD}\times 10^{9})/(1.56\times 10^{5}\times 10^{3}\mathrm{g tissue}).$$

#### Analysis of the Mitochondrial Membrane Potential

The alteration of mitochondrial membrane potential of intestinal epithelial cells of different treatment was measured spectrofluorometrically using Rhodamine 123 (Dash et al., 2014). Cells were seeded in six-well plates at a density of around 2 × 10^4^/well and incubated with 10 μl of 1.5 μM Rhodamine 123 at 37°C in the dark for 30 min. Then, fluorescence emitting from the Rh123 was measured for 2 min in a fluorescence spectrophotometer (Hitachi F-7000). The mitochondrial membrane potential was expressed as an emitting fluorescence level at an excitation wavelength of 493 nm and an emission wavelength of 522 nm.

#### DNA Fragmentation Study

The alkaline comet assay was done according to the method of Acharyya et al. (2015). A total of 75 ml of low melting point agarose (0.5%) in PBS at 37°C was added to a 25 ml cell suspension (∼10^5^ cells/ml). The mixture was then dropped onto a glass slide precoated with 1% agarose. The solidified slides were immersed in ice-cold lysis buffer (2.5 mM NaCl, 85 mM EDTA, 10 mM Trizma base, 1% Triton X-100, 10% DMSO, and 1% sodium lauryl sarcosinate, adjusted to pH 10.0) for 1 h. After lysis, the slides were repeatedly washed with PBS and placed in a submarine gel electrophoresis chamber filled with alkaline electrophoresis buffer (0.3 M NaOH and 1 mM EDTA). Then electrophoresis was done at 25 V and the current was adjusted to 300 mA. Slides were then neutralized with PBS and stained with a solution of 2 mg/ml ethidium bromide for 2 min. Slides were examined by fluorescence microscope.

### Statistical Analysis

Collected data were presented as the arithmetic mean (mean ± SD). The variations in different analysis results were examined by one-way ANOVA [least significant difference (LSD) testing]. A significant variation was accepted at the level of 5 and 1% (i.e., *p*< 0.05 and *p*< 0.01) was measured using Sigmastat 11.0 (United States) statistical software. Multiple correlations between the beneficial properties of the control, YAK, and BAK were performed by IBM-SPSS (version 19).

## Results and Discussion

India is a country which has not lost all of its culture, food habits, and traditions. Most ethnic people still prefer traditional food as a staple diet and these foods are commonly served to celebrate functions, marriages, and rituals. *Khambir* is a traditional flat bread prepared and consumed in the Ladhak region. The native people believe that it plays a health protective role in this extreme environment. However, this claim has not been scientifically validated thus far. Considering this, we evaluated its functional properties to justify its age-old health benefit claims at high altitudes.

### Total Count of Microbes

The microbial populations of *khambir* samples were examined and are represented in Table 1. There were no significant differences in the microbial content of mold, Bifidobacteria, *Vibrio* sp., *E. coli* between both types of the *khambir* samples (*P* ≤ 0.05). However, significant differences of LAB and yeast counts were observed between the YAK and BAK (*P* ≤ 0.05). The addition of yeast or butter milk as a starter in wheat flour leads to a profound microbial growth during the overnight incubation at room temperature and facilitated sourdough fermentation. Sourdough fermentation with LAB and yeasts leads to leavening and production of acid and CO_2_ in bread. The created anaerobiosis may have facilitated the growth of *Bifidobacterium* sp. in the dough. Surprisingly, the amount of mold was significantly higher which might be due to the fermented dough being wrapped with a wet cloth and the storage of the baked product in room temperature for several days. Fermented flat breads in the Indian cuisine have its own unique taste, way of preparation, and use of ingredients, and that leads to different microbial associations. *Selroti* from the Himalayan region (Yonzan and Tamang, 2010) and *ambali* from South India (Ramakrishnan, 1980) contain mostly LAB. Whereas, wheat-based bread like *Bhatooru* from Himachal (Savitri and Bhalla, 2013), *seera* from the Middle and North of India (Savitri et al., 2012), and *nan* (Batra, 1986) is dominated by yeasts and very meager populations of LAB. The presence of *Vibrio* sp. in *khambir* is of great concern for its hygienic status which may be related to the quality of water (as the native people in the Himalayan region use glacier water directly for household purpose). However, the pathogenic property of an organism depends on the strain and host specificity as reflected by the regular consumption of such breads by the native people of the Himalayan region. Tamang et al. (2015) mentioned that about 80% of traditional fermented foods that are prepared through natural fermentation may contain functional, non-functional, and pathogenic microorganisms. The prevalence of pathogenic bacteria such as *S. aureus*, *Bacillus cereus*, *E. coli*, *Campylobacter*, *Vibrio cholerae, Aeromonas*, *Klebsiella*, *Shigella* sp., and *Salmonella* among others in traditional fermented foods was also documented by Abriouel et al. (2017). Additionally, microbial interplay (enzymes and metabolites) during the course of sourdough fermentation can delay starch digestibility leading to lower glycemic responses, reduced gluten content, and other antinutrients, modulates accessibility of bioactive components, and improves mineral bioavailability, thus enhancing gut health (Poutanen et al., 2009).

**Table 1:** Enumeration of different group of microbes in both YAK and BAK.

	Microbial *composition* (log_10_ cfu/g)								
	Total aerobic	Yeast	Mold	LAB	*Bifidobacterium* sp.	*E. coli*	*Salmonella* sp.	*Vibrio* sp.
YAK	8.90 ± 0.33	7.96 ± 0.82	6.30 ± 1.22	1.32 ± 0.14	2.86 ± 0.49	5.30 ± 1.44	0	3.30 ± 0.28
BAK	11.64 ± 0.77	3.30 ± 0.46	6.60 ± 0.76	2.4 ± 0.13	3.23 ± 0.86	5.77 ± 1.74	0	3.77 ± 0.24

### Phenolic and Flavonoid Content of *Khambir*

It was estimated that the phenolic content of YAK and BAK was 2.37 and 1.29 mg/g, respectively (Table 2), which is a much higher value than the unfermented or unprocessed wheat flour of Indian varieties (Punia and Sandhu, 2016) as well as Chinese varieties (Li et al., 2015). Additionally, a significant or comparatively higher amount of phenolic acids like protocatechuic acid, *p*-hydroxy-benzoic acid, *p*-coumaric acid, and ferulic acid were also present in the YAK and BAK (Table 2). Similarly, flavonoid content in the fermented leavened bread was also increased many fold (1.63–2.23 mg/g in the *khambir* and 80–100 mg/g in unfermented wheat flour as reported by Punia and Sandhu, 2016]. During fermentation, microbes originating from hydrolytic enzymes (cellulases, esterases, glycosidase, polyphenol hydrolase, etc.) may lead to the branching and defabrication of the cellulosic backbone as well as polyphenolic structures; therefore, phenolics and flavonoid compounds are detached from the anchoring molecule and become free. Dietary flavonoids and phenolic acids have attracted much interest recently because they have a variety of beneficial biological properties and play an important role in the protection and prevention of many human diseases (Jalal et al., 2015).

**Table 2:** Phenolics and flavonoid content in *khambir* samples.

	Total phenolics (mg/g)	Protocatechuic acid (mg/kg)	p-Hydroxy-benzoic acid (mg/kg)	p-Coumaric acid (mg/kg)	Ferulic acid (mg/kg)	Sinapic acid (mg/kg)	Total flavonoids (mg/g)
YAK	2.37 ± 0.21	18.31 ± 0.46	13.87 ± 0.57	2.26 ± 0.23	16.42 ± 0.82	ND	2.23 ± 0.4
BAK	1.29 ± 0.2	16.52 ± 0.43	7.53 ± 0.60	1.34 ± 0.33	18.34 ± 1.68	ND	1.60 ± 0.2

*Data are presented as means ± standard deviation of three replicates.*

### Antimicrobial Activity of Water Extract of *Khambir*

The antimicrobial activity of the aqueous extracts of *khambir* samples was tested against different strains of enteropathogens and other organisms (Table 3). It was found that YAK exerted more strain specific antibiosis, particularly against enteropathogens like *S. typhi*, *S. dysenteriae*, *S. faecalis*, and *V. harveyi* than BAK. The YAK also showed significant inhibitory effects against *S. aureus*, whereas the killing effect of BAK was more prominent against *M. luteus.* The results indicated that the reactive metabolites that evolved during fermentation act as a natural preservative in this food. The strain specific and variable antimicrobial effect of natural medicines is a common phenomenon and many factors like pH, extracting solvent and techniques, dilution, culturing media, and source of microorganism are very important and can alter the interaction of the active ingredients with medicinal flora (Rios and Recio, 2005). The antibiosis of the tested food samples was very significant and comparable with commercial antibiotics like tetracycline, which is commonly used as a food preservative (Table 3).

**Table 3:** Antimicrobial activity of aqueous extract of two types of *khambir* products.

Target microbes	YAK (zone of inhibition, mm)	AI	BAK (zone of inhibition, mm)	AI
*Aeromonas hydrophila* SBK1	ND	–	ND	–
*Salmonella typhi* B3274				
	6.5 ± 0.6,	0.56	ND	–
*Salmonella typhi* E1590				
	6.0 ± 0.6	0.36	3.5 ± 0.3	0.21
*Salmonella typhi* MTCC 734	5.5 ± 0.4	0.47	4.5 ± 0.4	0.39
*Shigella dysenteriae* 4717				
	5.2 ± 1.04	0.46	5.5 ± 0.5	0.49
*Shigella sonnei* RS 1				
	ND	–	ND	–
*Staphylococcus aureus* MB 13				
	7.5 ± 0.8	0.63	ND	–
*Streptococcus faecalis* MB 15				
	6.2 ± 0.6	0.65	ND	–
*Micrococcus luteus* ATCC 9341				
	7.0 ± 0.6	0.56	7.2 ± 0.7	0.58
*Vibrio harveyi* MTCC 7954				
	8.5 ± 0.8	0.94	6.5 ± 0.7	0.76

*Data are presented as means ± standard deviation of three replicates. Activity index (AI) = inhibitory zone of test sample (excluding disc diameter, 5 mm)/inhibitory zone of a standard drug (tetracycline, 10 mg/disc).*

### β-Glucosidase (BG), Acetylcholine Esterase (AChE), and Trypsin Inhibitory Potentialities of *Khambir* Extracts

**FIGURE 2:**
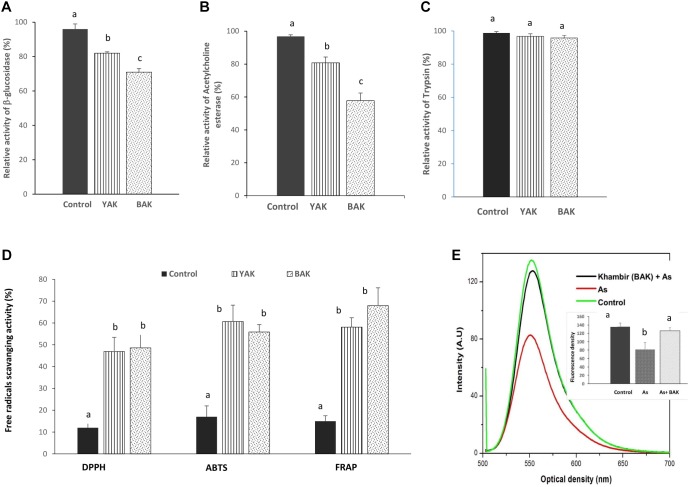
Evaluation of bioactivities of aqueous extract of yeast added *khambir* (YAK) and buttermilk added *khambir* (BAK). Changes of β-glucosidase **(A)**, acetylcholine esterase **(B)**, trypsin **(C)** inhibitory activities, and *in vitro* antioxidant **(D)** activities (DPPH, ABTS, FRAP) of fermented *khambir*, i.e., YAK and BAK in respect to control (unfermented) were determined. The activity of enzymes (without any additive) was considered as 100%. **(E)** The alteration of mitochondrial transmembrane potential in respect to emitted fluorescence level of Rhodamine 123 during exposure of arsenic and arsenic + *khambir* extract (BAK) in *in situ* intestinal loop model study. The control is indicated the fluorescence intensity of control tissue. Data presented as the mean ± standard deviation of five replicates. Different superscripts (alphabet) on the bar indicated the level of significance difference (*p* < 0.05) among respective groups.

Glucosidase inhibitors have significant therapeutic potential in the treatment of metabolic diseases and disorders like diabetes, obesity, human immune deficiency virus infection, metastatic cancer, lysosomal storage disease, etc. (Pandey et al., 2013). Over 100 glycosidase inhibitors have been isolated from plants and microorganisms (Pandey et al., 2013). A group of microbes originating from β-glucosidase inhibitors like acarbose, voglibose, valienamine, adiposin-1, and trestatin-B are commercially exploited as anti-diabetic drugs which can reduce sugar digestion (undigested resistant starch) and assimilation into the body (Kulkarni-Almeida et al., 2011). Both *khambir* preparations, YAK and BAK exhibited 18 and 29% inhibition of β-glucosidase activity (Figure 2A), establishing them as a useful diabetic diet. Our experimental results provide clues for the blood sugar lowering abilities of sourdough bread as it has previously been reported that sourdough bread consumption can lower post-prandial blood glucose and improve insulin and GLP-1 responses in human subjects (Maioli et al., 2008). In addition, the aqueous extract of YAK and BAK also exerted anti-AChE activity by inhibiting 19.2 and 42.2% of the original activity (Figure 2B). The occurrence of AChE inhibitors in natural resources has been well documented and characterized, but the quest for new inhibitors remains crucially important owing to their therapeutic potential in the treatment of neurological disorders such as Alzheimer’s disease, senile dementia, ataxia, myasthenia Gravis, and Parkinson’s disease (Elumalai et al., 2015). The potentialities of BAK have been proven to be more profound, with respect to the inhibition of β-glucosidase and AChE activity, than YAK, and this may relate to the abundance of a consortium of reactive metabolites particularly phenolic constituents in the *khambir* product.

**FIGURE 3:**
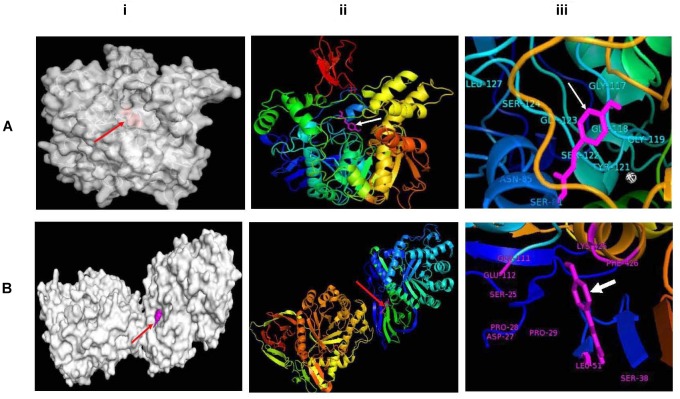
Molecular docking between p-coumaric acid with acetylcholine esterase **(A)** and β-glucosidase **(B)**. Docked proteins have been shown as gray surface models (i); proteins have been visualized as ribbons docked with p-coumaric acid (stick, magenta) (ii); and docked p-coumaric acid (stick, magenta) at the active sites of proteins (iii).

Together with the experimental results and statistical relationship, molecular docking experiments were performed for a better understanding of how the phenolic compound especially *p*-coumaric acid interacts with both the AChE and BG enzyme. In the presence of certain functional groups, such as hydroxyl, carboxyl, and acrylic acid groups, *p*-coumaric acid can act as a hydrogen bond acceptor or donor, which seems to increase the potency of inhibiting the activity of AChE and BG. Molecular docking analysis showed that *p*-coumaric acid has an optimum binding affinity (ΔG of -6.8 kcal/mol) with a molecular target in the second cavity of the predicted active sites of AChE and the amino acids in this site of the enzyme like SER-81, ASN-85, GLY-117, GLY 118, GLY-119, TYR-121, SER-122, GLY-123, SER-124, and LEU-127 formed H-bonds with the phenolic compound (Figure 3A). On the other hand, *p*-coumaric acid has blocked the activity of BG by possibly binding at the third cavity of the predicted active sites with an optimum binding energy or binding affinity of -7.6 kcal/mol. This interaction occurs via amino acids like ASP-27, PRO-28, PRO-29, SER-38, LEU-51, GLU-111, GLU-112, LYS-425, and PHE-426 at the active site (Figure 3B). The calculated absolute binding free energies in between -6.8 to -7.6 kcal/mol indicated that a number of relatively weak chemical interactions (non-covalent bonds) stabilize the conformations and the interactions between the molecules. This result clearly documents that a functional component of *khambir* is phenolics, which can specifically bind and inhibit the activities of both BG and AChE. A similar pattern of interaction for the inhibition of angiotensin-converting enzyme with the phenolic acids was also observed by Shukor et al. (2013).

Both extracts of *khambir* were non-responsive to trypsin activity (Figure 2C). A reduction in trypsin inhibitory activity during natural lactic acid fermentation of cereals was reported by Osman (2011). This indicates that the *khambir* has no such adverse effect on protein digestion.

### Assay of *in vitro* Antioxidant Activity

Recently, there has been increased interest on antioxidant nutrients, which have the ability to scavenge free radicals in the system and neutralize them before they do any damage to body cells. A number of methods are also available to determine *in vitro* antioxidant activity each with their own specific reaction principles; therefore, researchers employ many methods simultaneously to obtain a clear picture of the antioxidant activity. In this study, DPPH, ABTS, and ferric reducing/antioxidant power (FRAP) methods were employed to evaluate the antioxidant potentialities of the aqueous extract of *khambir*.

The activity of DPPH, ABTS, and FRAP of control (unfermented), YAK, and BAK are shown in Figure 2D. The DPPH, ABTS, and FRAP activity of the control sample was 12, 17, and 15%; for YAK was 46.90, 60.67, and 58.11%; and for BAK was 48.62, 55.85, and 67.92%, respectively (Figure 2D). This result indicated that antioxidative potentialities of wheat flour were significantly (*p* < 0.05) improved due to fermentation by a consortium of microbes.

The “antioxidant power” of the *khambir* is greatly related to its high phenolic content, as these molecules have the innate ability to donate a hydrogen or electron. They have the ability to delocalize the unpaired electron of free radicals within the aromatic structure (Jalal et al., 2015; Li et al., 2015; Punia and Sandhu, 2016), thereby exhibiting various physiological activities including anti-inflammatory, antiallergic, anticarcinogenic, antihypertensive, anti-arthritic, and antimicrobial activities.

### Antioxidant and Antitoxic Action of *Khambir*: *In situ* Experiment in Rat Intestine

The activities of SOD and CAT in the control and experimental groups are shown in Table 4. Arsenic-induced depletion (*p* < 0.01) of SOD and CAT activity was found in the respective group compared to the controls. Co-supplementation of the *khambir*, along with arsenic, significantly (*p* < 0.05) elevated the levels of SOD and CAT compared to the arsenic alone-exposed groups.

**Table 4:** Activity of antioxidant defense related biomarkers in intestinal epithelia during exposure of arsenic.

Group	SOD (U)	Catalase (U)	MDA (nM/g)	GSH (mg/g)
Untreated control	1.72 ± 0.03	1.87 ± 0.07	119.42 ± 6.65	31.43 ± 1.27
*Khambir*	1.75 ± 0.12	1.92 ± 0.06	92.46 ± 4.34^∗^	31.87 ± 2.83
Arsenic	0.64 ± 0.04^∗∗^	1.19 ± 0.06^∗∗^	165.24 ± 6.2^∗∗^	49.82 ± 5.86^∗∗^
YAK + arsenic	1.17 ± 0.04^∗^	1.55 ± 0.05	104.42 ± 5.72	38.43 ± 5.66^∗^
BAK + arsenic	1.62 ± 0.03	1.85 ± 0.05	96.04 ± 3.45^∗^	35.19 ± 4.11

*Data are presented as means ± standard deviation (n = 5). ^∗^p < 0.05 and ^∗∗^p < 0.01 are the level of significance difference compared with the control group.*

The administration of *khambir* alone diminished the level of MDA formation compared to normal levels in intestinal tissues. Arsenic treatment resulted in a significant (*p* < 0.01) elevation of the MDA level compared to the control group. Administration of *khambir* (YAK and BAK), along with arsenic, antagonized the toxic effects of arsenic that were reflected by the significant (*p* < 0.05) decrease of MDA levels compared to the arsenic alone-exposed groups (Table 4).

The content of GSH increased about 36% (*p* < 0.05) in the intestinal epithelia when exposed to arsenic compared to the control. Simultaneous *khambir* (both YAK and BAK) treatment with arsenic significantly (*p* < 0.05) decreased the GSH level in the tissue compared to the arsenic alone-treated groups (Table 4).

Arsenic toxicity leads to the disruption of the mitochondrial membrane architecture, which is reflected by the significant reduction (*p* < 0.05) of the mitochondrial transmembrane potential compared to the control (Figure 2E). Mitochondrial membrane permeability disruption is associated with a lack of rhodamine 123 retention and a decrease in fluorescence. *Khambir* extract protects the mitochondrial membrane from arsenic toxicity which was noted by the level of fluorescence intensity near the control level (Figure 2E).

**FIGURE 4:**
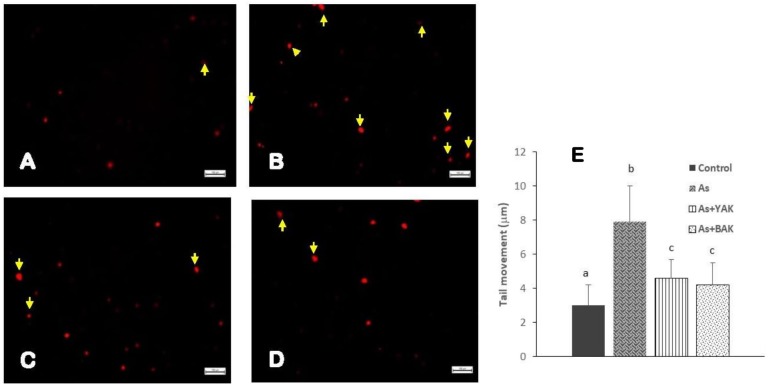
Determination of the genotoxic protective effects of *khambir* extracts against arsenic toxicity by Comet assay. Fluorescent microscopic image of **(A)** control tissue, **(B)** arsenic treated, **(C)** co-supplementation of arsenic and YAK, and **(D)** co-supplementation of arsenic and BAK; **(E)** graphical representation of the comet tail length of different experiments. The comet tail length was calculated as the distance between the end of nuclei heads and end of each tail. Values are expressed as the mean ± SD (*n* = 100); different superscripts (alphabet) on the bar indicate the different levels of significance (*p* < 0.05) among respective groups.

The comet assay was carried out to measure the single-strands DNA breaks in the intestinal epithelial cells of the control, arsenic treatment, and arsenic along with *khambir* treatment (Figure 4). Results showed an enhanced number of tail migration (DNA strand break) in the arsenic-treated group, which was significantly restrained in the *khambir* supplemented arsenic-exposed group. The extrusion of the damaged DNA from the majority of cells in the arsenic treated group was clearly visualized (morphometric analysis) by noting the comet tail length (Figure 3). Supplementation of *khambir* extracts antagonized the toxic effects of arsenic, resulting in a lower amount of DNA damage as well as comet tail length as compared to arsenic alone.

*Khambir*, particularly BAK extract, exhibited strong protection against arsenic induced modification of the enzymatic antioxidant defense system, by restoring the activities of SOD and CAT, preventing LPO, restoring the GSH pool and mitochondrial transmembrane potential, and above all preventing DNA fragmentation from the harsh toxic effects of arsenic. *Khambir* extract, particularly its phenolic constituents, may alleviate arsenic toxicity by means of its antioxidant components via a number of mechanisms, including the protection of target molecules (lipids, proteins, and nucleic acids) from oxidative damage (by neutralizing the generated free radicals), suppressing the inflammatory response, modulating vascular homeostasis, and improving the cellular defense system by altering the expression at the gene level (Jalal et al., 2015; Li et al., 2015). The dietary fiber (1.2 g%) of *khambir* may also play a pivotal role in detoxification by entrapping arsenic before exerting any toxicity (Abdel-Salam et al., 2010).

### Multiple Correlation

Table 5 shows the correlation coefficient (*r*) between total phenolic, flavonoid, phenolic acids, inhibition of AChE and β-glucosidase, *in vitro* antioxidant activities, and *in vivo* antioxidant parameters altered in arsenic treated rats during supplementation of BAK. The phenolics and flavonoids have a strong relationship with the parameters like *in vitro* and *in vivo* antioxidant properties, and inhibition of AChE and β-glucosidase activities as an *r*-value lies in between +1 to -1. A strong positive correlation was observed between total phenolics, total flavonoids, protocatechuic acid, *p*-hydroxy-benzoic acid, ferulic acid, *p*-coumaric acid, DPPH, ABTS, FRAP, SOD, and CAT. Among the phenolics, *p*-coumaric acid exhibited a strong position correlation with the inhibition of AChE and β-glucosidase. In contrast, a negative correlation was obtained between the phenolics and the content of GSH and MDA.

**Table 5:** Multiple correlation test among the phenolic, *in vitro* and *in vivo* antioxidant profiles, and other health beneficial effects.

	Total phenolics	Total flavonoids	Protocatechuic acid	p-hydroxy-benzoic acid	p-coumaric acid	Ferulic acid	ACE	BG	DPPH	ABTS	FRAP	SOD	Catalase	MDA	GSH
Total phenolics	1	0.956	0.895	0.992	0.872	0.675	−0.282	−0.442	0.765	0.849	0.673	0.423	0.427	−0.718	−0.644
Total flavonoids		1	0.987	0.911	0.689	0.862	−0.552	−0.687	0.921	0.967	0.861	0.671	0.675	−0.892	−0.841
Protocatechuic acid			1	0.832	0.562	0.933	−0.680	−0.796	0.972	0.995	0.932	0.782	0.786	−0.953	−0.918
p-Hydroxy-benzoic acid				1	0.927	0.576	−0.159	−0.326	0.678	0.775	0.574	0.305	0.310	−0.625	−0.543
p-Coumaric acid					1	0.227	0.224	0.053	0.352	0.481	0.224	−0.075	−0.070	−0.286	−0.188
Ferulic acid						1	−0.898	−0.960	0.992	0.963	1.000	0.954	0.956	−0.998^∗^	−0.999^∗^
ACE							1	0.985	−0.834	−0.747	−0.900	−0.989	−0.988	0.870	0.915
BG								1	−0.916	−0.850	−0.961	−1.000	−1.000	0.942	0.971
DPPH									1	0.990	0.991	0.907	0.909	−0.998^∗^	−0.985
ABTS										1	0.962	0.838	0.841	−0.978	−0.951
FRAP											1	0.955	0.957	−0.998^∗^	−0.999^∗^
SOD												1	1.000	−0.934	−0.965
Catalase													1	−0.936	−0.967
MDA														1	0.995
GSH															1

*^∗∗^Correlation is significant at p < 0.01 and ^∗^correlation is significant at p < 0.05.*

## Conclusion

This study clearly demonstrated that both types of *khambir*, YAK and BAK, carry health benefits and are rich in phenolics, as they exhibit significant antimicrobial, antioxidant, antiradical, and anti-toxic effects. Additionally, a group of food graded microbes in the product can provide some added advantages to consumers. Thus, the experimental evidence supports its age-old claim as a healthy and protective food source against environmental stresses. Further, scientific intervention is urgently needed to improve its hygienic status which will expand its market demand as well as pave the way for economic and livelihood development of the ethnic people of the Leh-Ladakh regions.

## Ethics Statement

This study was carried out in accordance with the recommendations of Vidyasagar University Animal Ethical clearance (ICE/7-8/6-8/16 dt. 26.08.2016) with written informed consent from all subjects. The study and protocol were reviewed and approved by the Institutional Ethics Committee (IEC) of Vidyasagar University.

## Author Contributions

The trial was conceived by PH, MR, SM, PD, and KM. KM designed the study. PH, MR, and SM conducted the research. SP, MT, KG, and PH analyzed the data. DB, SS, SD, and KM prepared the manuscript. KM had the primary responsibility of the final content. All authors read and approved the final manuscript.

## Conflict of Interest Statement

The authors declare that the research was conducted in the absence of any commercial or financial relationships that could be construed as a potential conflict of interest.

## Footnotes

1 http://sts.bioe.uic.edu/castp/

2 https://pubchem.ncbi.nlm.nih.gov/
